# Supplementary material for: Quantized resistance revealed at the criticality of the quantum anomalous Hall phase transitions
Source: Nat Commun. 2023 Sep 9;14:5558. doi: 10.1038/s41467-023-40784-y (PMC10492779; doi:10.1038/s41467-023-40784-y)
Supplement: Supplementary file 2 — Supplementary Information [file 41467_2023_40784_MOESM2_ESM.pdf]

Supplementary Information

Quantized resistance revealed at the criticality of the quantum anomalous Hall phase transitions

Peng Deng[1*], Peng Zhang[1], Christopher Eckberg[2,3,4], Su Kong Chong[1], Gen Yin[1], Eve Emmanouilidou[5], Xiaoyu Che[1], Ni Ni[5], and Kang L. Wang[1,5*]

[1]*Department of Electrical and Computer Engineering, University of California Los Angeles, Los Angeles, California 90095, USA*

[2]*Fibertek Inc, Herndon, VA 20783, USA*

[3]*US Army Research Laboratory, Adelphi, MD 20783, USA*

[4]*US Army Research Laboratory, Playa Vista, CA 20783, USA*

[5]*Department of Physics and Astronomy, University of California Los Angeles, Los Angeles, California 90095, USA*

[*]Corresponding author. Email: wang@ee.ucla.edu(K.L.W), dengpeng@g.ucla.edu (P.D.)

1. QAH-NI transition: the opposite field sweeping direction.

In Fig. 2 in the main text, quantized values of ρ_{xx} and σ_{xy} are revealed at the criticality of the QAH insulator to normal insulator phase transition when the magnetic field is swept from negative to positive field. Supplementary Fig. 1 presents the result for the opposite sweeping direction. As can be seen, when plotted against B^* (= $B - \mu_0 H_c$), all ρ_{xx} curves measured at different temperatures converge at a single point with a quantized value of h/e^2 (Supplementary Fig. 1a,1b). Meanwhile, besides the origin point, all σ_{xy} curves converge at a single point with a quantized value of 0.5 e^2/h (Supplementary Fig. 1c,1d). Such results show that the values of ρ_{xx} and σ_{xy} at the criticality are universal among different QAH insulator to normal insulator phase transitions within a single sample.

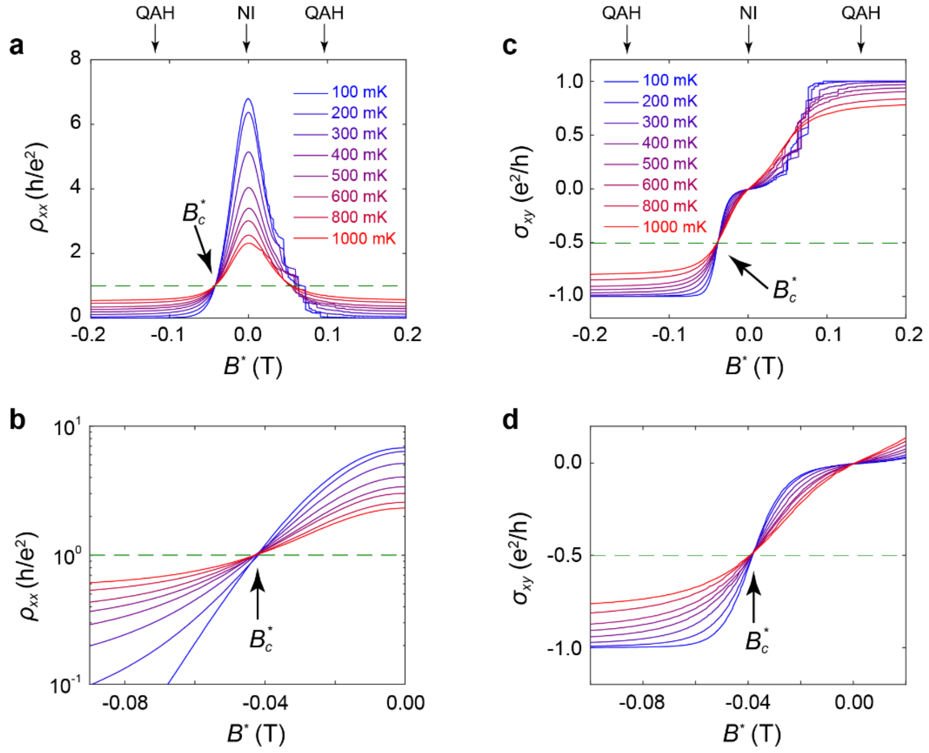

Supplementary Figure 1. a, B^* (= $B - \mu_0 H_c$) dependence of ρ_{xx} under different temperatures. All curves converge to a single point at B_c^* with a quantized value of h/e^2. **b,** Zoom-in plot of **a** at around B_c^*. **c,** B^* (= $B - \mu_0 H_c$) dependence of σ_{xy} under different temperatures. All curves converge at B_c^* with a quantized value of 0.5 e^2/h. **d,** Zoom-in plot of **c** at around B_c^*.

2. QAH-AXI transition: the opposite field sweeping direction.

Similar to the QAH insulator to normal insulator phase transition case, the values of ρ_{xx} and σ_{xy} are also universal among different QAH insulator to axion insulator phase transitions that occurred in a single sample. In Fig. 3 and Fig. 4 in the main text, the V and Cr transitions occur when the field is swept from positive to negative. Supplementary Fig. 2 is the results measured in the same sample but with the opposite field sweeping direction. For both the V transition (Supplementary Fig. 2a-2c) and the Cr transition (Supplementary Fig. 2d-2f), quantized values of ρ_{xx} and σ_{xy} are obtained at the critical point, confirming the universalness of the critical values.

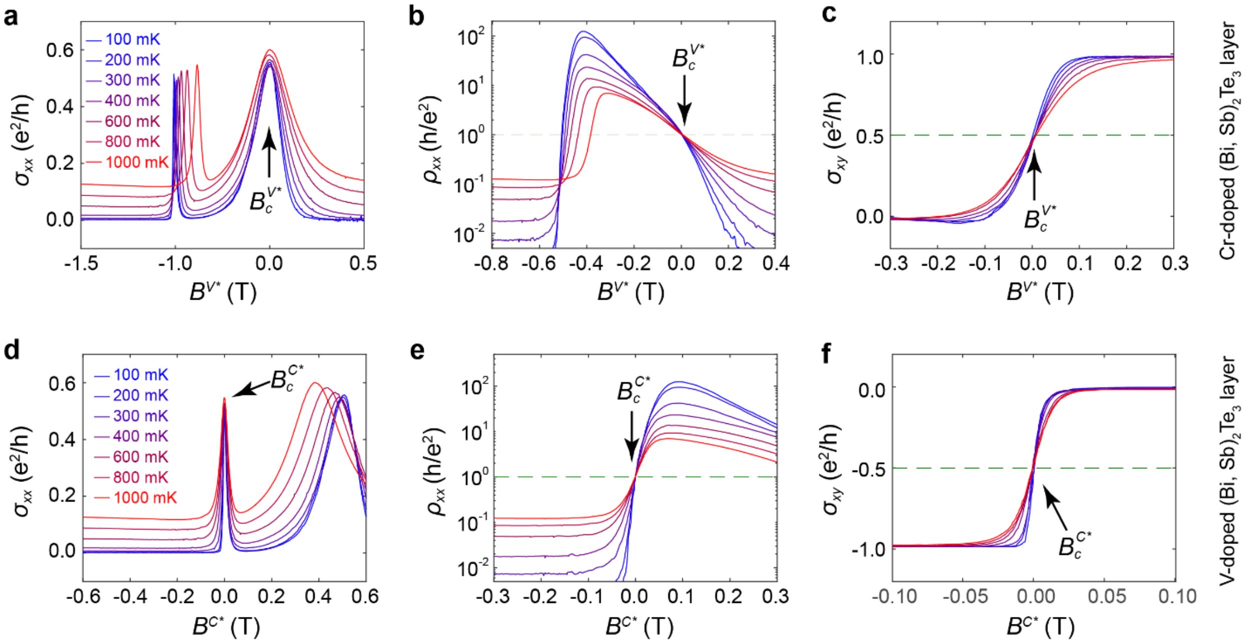

Supplementary Figure 2. a-c, B^{V*} (= $B - \mu_0 H_c^V$) dependence of σ_{xx}, ρ_{xx}, and σ_{xy} under different temperatures, respectively. At $B^{V*} = B_c^{V*}$, the values of critical ρ_{xx} and σ_{xy} are quantized. **d-f,** B^{C*} (= $B - \mu_0 H_c^C$) dependence of σ_{xx}, ρ_{xx}, and σ_{xy} under different temperatures, respectively. At $B^{C*} = B_c^{C*}$, the values of critical ρ_{xx} and σ_{xy} are quantized.

3. QAH-NI transitions in other samples

The above results show that the critical values of ρ_{xx}, and σ_{xy} are universal among different transitions within a single sample. To testify whether this universalness exists across different samples, we first examined the QAH-NI transitions in two more Cr-doped (Bi, Sb)$_2$Te$_3$ samples,

and the results are shown in Supplementary Fig. 3 and 4. Again, ρ_{xx}^{c} = h/e^2 and σ_{xy}^{c} = 0.5 e^2/h are obtained for both samples, indicating it is a universal phenomenon insensitive to sample details.

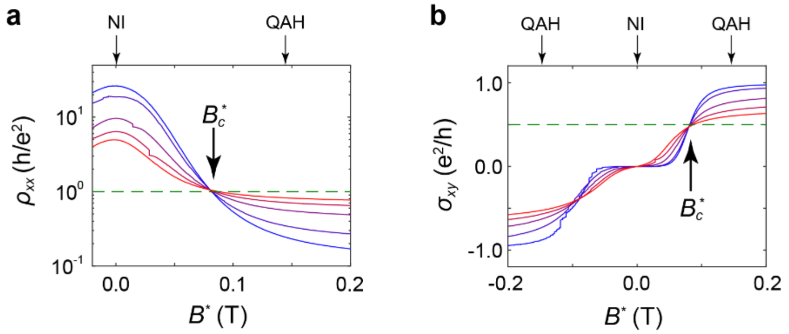

Supplementary Figure 3. B^* (= $B - \mu_0 H_c$) dependences of **a,** ρ_{xx}, and **b,** σ_{xy} for Cr-doped (Bi, Sb)$_2$Te$_3$ sample #2.

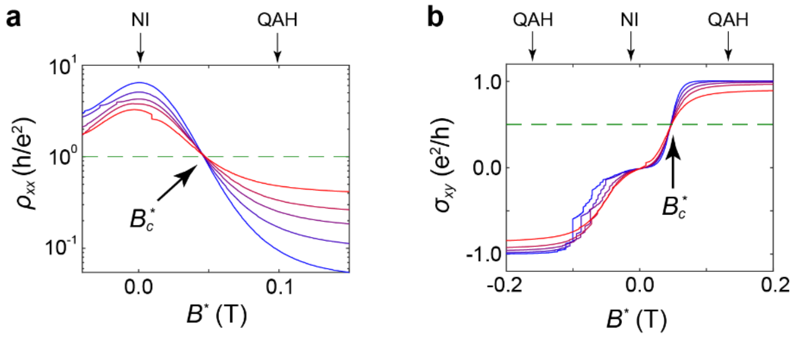

Supplementary Figure 4. B^* (= $B - \mu_0 H_c$) dependences of **a,** ρ_{xx}, and **b,** σ_{xy} for Cr-doped (Bi, Sb)$_2$Te$_3$ sample #3.

4. QAH-AXI transitions in other samples

Similarly, the universalness of the critical values in the QAH-AXI transitions is also testified across multiple Cr-doped (Bi, Sb)$_2$Te$_3$/ (Bi, Sb)$_2$Te$_3$/ V-doped (Bi, Sb)$_2$Te$_3$ samples, and the results are shown in Supplementary Fig. 5 and Fig. 6. For the two additional AXI samples studied here, we examined both the Cr transition and V transition. As can be seen, quantized ρ_{xx}, and σ_{xy} are revealed in the four transitions of these two samples. In short, all of the QAH-NI and QAH-AXI transitions show quantized values of the transport coefficients at the critical point, confirming the reproducibility of our results and the robustness of the new analysis protocol.

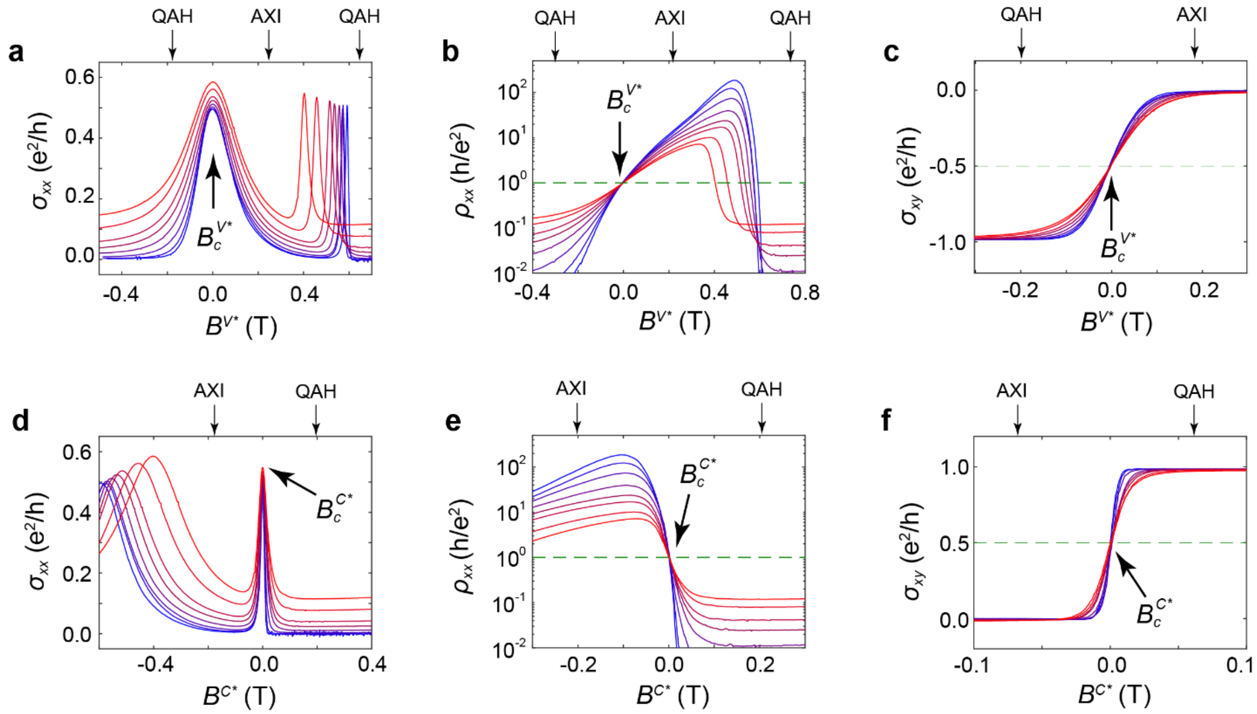

Supplementary Figure 5. **a-c**, B^{V*} (= $B - \mu_0 H_c^V$) dependence of σ_{xx}, ρ_{xx}, and σ_{xy}, respectively, under different temperatures for Cr-doped (Bi, Sb)$_2$Te$_3$/ (Bi, Sb)$_2$Te$_3$/ V-doped (Bi, Sb)$_2$Te$_3$ sample #2. **d-f**, B^{C*} (= $B - \mu_0 H_c^C$) dependence of σ_{xx}, ρ_{xx}, and σ_{xy} for the same sample.

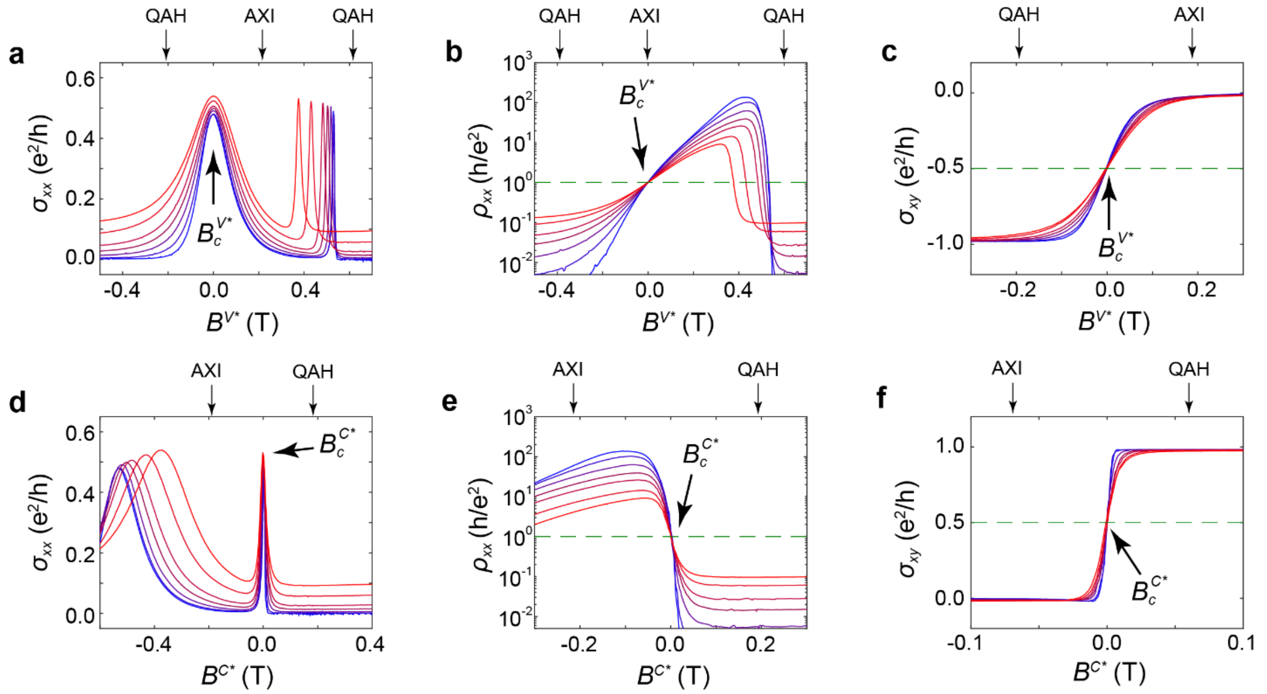

Supplementary Figure 6. **a-c**, B^{V*} (= $B - \mu_0 H_c^V$) dependence of σ_{xx}, ρ_{xx}, and σ_{xy}, respectively, under

different temperatures for Cr-doped (Bi, Sb)$_2$Te$_3$/ (Bi, Sb)$_2$Te$_3$/ V-doped (Bi, Sb)$_2$Te$_3$ sample #3. **d-f**, B^{C*} (= $B - \mu_0 H_c^C$) dependence of σ_{xx}, ρ_{xx}, and σ_{xy} for the same sample.

5. Critical exponent in QAH-NI transitions

Supplementary Fig. 7a presents the field dependence of σ_{xy} for a 6 QL Cr-doped (Bi, Sb)$_2$Te$_3$. By first sweeping the field from right to left then backward, four QAH-NI transitions, Cr left (1), Cr left (2), Cr right (1), and Cr right (2), are revealed in sequence in a hysteresis loop. Among these transitions, discrete jumps can be found in Cr left (1) and Cr right (1), these discontinuity makes them not suitable for scaling analysis. Therefore, we only focus on the transitions at the higher fields, and the results are displayed in Supplementary Fig. 7b. As can be seen, critical power-law temperature dependences with consistent values of the critical exponents are revealed in both transitions.

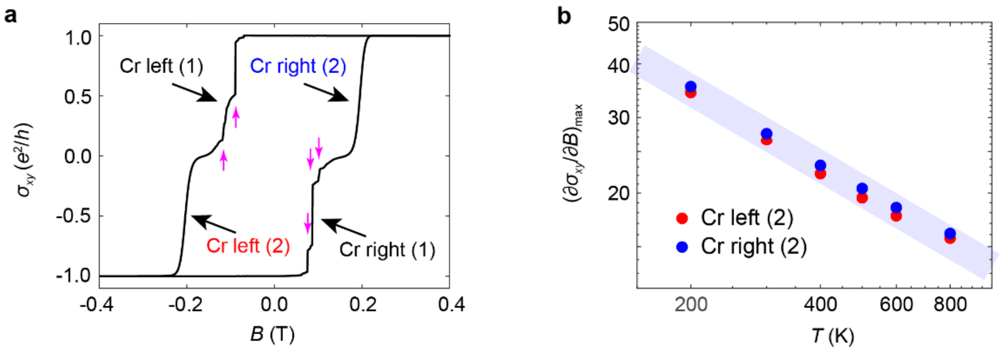

Supplementary Figure 7. **a**, Field dependence of σ_{xy} for the 6 QL Cr-doped (Bi, Sb)$_2$Te$_3$. Four QAH-NI transitions (black arrows) are shown in the hysteresis loop. The discrete jumps in the two inner transitions are highlighted by magenta arrows. **b**, Temperature dependent $(\partial\sigma_{xy}/\partial B)_{max}$ for the Cr left (2) and Cr right (2) transitions.

6. The one-step magnetization reversal and the origin of zero Hall plateaus in Cr-doped (Bi, Sb)$_2$Te$_3$

As shown in Fig. 1 in the main text, both the 6 QL Cr-doped (Bi, Sb)$_2$Te$_3$ and the Cr-(Bi, Sb)$_2$Te$_3$/(Bi, Sb)$_2$Te$_3$/V-doped (Bi, Sb)$_2$Te$_3$ heterostructure exhibit zero Hall plateaus during the

magnetization reversal process. Despite their similar transport features, these two system have distinct origins for their observed zero Hall plateaus. In the case of heterostructure, the magnetization reversal occurs in a "two-step" process, and the zero Hall plateaus arise from the axion insulating state when the magnetization in top and bottom magnetic layers are antiparallelly aligned. Conversely, in the Cr-doped (Bi, Sb)$_2$Te$_3$, the magnetization reversal is a "one-step" process and the zero Hall plateaus appears when the sample enters the NI phase as the hybridization gap exceeds magnetization gap.

To demonstrate that the magnetization reversal in Cr-doped (Bi, Sb)$_2$Te$_3$ is not a "two-step" process as in the AXI sample, we grew an 8 QL Cr-doped (Bi, Sb)$_2$Te$_3$ sample, which is thicker than the 6 QL sample studied in the main text. In the 8 QL sample, the hybridization gap is vanishing. As a result, the NI is absent during the magnetization reversal process, and the zero Hall plateaus are absent as well, as shown in Supplementary Fig. 7.

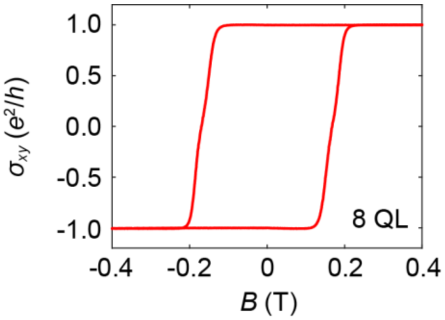

Supplementary Figure 8. Field dependence of Hall conductance for an 8 QL thick Cr-doped (Bi, Sb)$_2$Te$_3$ sample.

7. A summary of the critical values reported in the phase transitions of QAH insulators

Supplementary Table 1. Critical values in QAH phase transitions

Sample	Transition	Critical values	Ref.
4 QL V-doped (Bi,Sb)$_2$Te$_3$	QAH-Anderson insulator	ρ_{xx} ~ 1.15 h/e^2	1
Cr-doped (Bi,Sb)$_2$Te$_3$/(Bi,Sb)$_2$Te$_3$/Cr-doped (Bi,Sb)$_2$Te$_3$	QAH-NI	ρ_{xx} ~ h/e^2<br>σ_{xy} ~ 0.44 e^2/h	2

Cr-doped $(Bi,Sb)_2Te_3$/$(Bi,Sb)_2Te_3$/Cr-doped $(Bi,Sb)_2Te_3$	QAH-NI	ρ_{xx} ~ 1.3 h/e^2<br>σ_{xy} ~ 0.44 e^2/h	3
Cr-doped $(Bi,Sb)_2Te_3$	QAH-NI	ρ_{xx} ~ 2.6 h/e^2	4
V-doped $(Bi,Sb)_2Te_3$/$(Bi,Sb)_2Te_3$/Cr-doped $(Bi,Sb)_2Te_3$	QAH-AXI	ρ_{xx} ~ h/e^2 (sample 1)<br>ρ_{xx} ~ 1.1 h/e^2 (#2)<br>ρ_{xx} ~ 2.1 h/e^2 (#3)	5
6 septuple layers $MnBi_2Te_4$	QAH-AXI	ρ_{xx} ~ 0.85 h/e^2	6

References

1 Chang, C.-Z. *et al.* Observation of the quantum anomalous Hall insulator to Anderson insulator quantum phase transition and its scaling behavior. *Phys. Rev. Lett.* **117**, 126802 (2016).

2 Kawamura, M. *et al.* Topological quantum phase transition in magnetic topological insulator upon magnetization rotation. *Phys. Rev. B* **98**, 140404 (2018).

3 Kawamura, M. *et al.* Current scaling of the topological quantum phase transition between a quantum anomalous Hall insulator and a trivial insulator. *Phys. Rev. B* **102**, 041301 (2020).

4 Liu, C. *et al.* Distinct quantum anomalous Hall ground states induced by magnetic disorders. *Phys. Rev. X* **10**, 041063 (2020).

5 Wu, X. *et al.* Scaling behavior of the quantum phase transition from a quantum-anomalous-Hall insulator to an axion insulator. *Nat. Commun.***11**, 4532 (2020).

6 Liu, C. *et al.* Robust axion insulator and Chern insulator phases in a two-dimensional antiferromagnetic topological insulator. *Nat. Mater.* **19**, 522-527 (2020).
